# ARHGAP40 suppresses clear cell renal cell carcinoma progression and is associated with altered glutamine-related metabolism and redox balance

**DOI:** 10.1080/15384047.2026.2746137

**Published:** 2026-10-08

**Authors:** Na You, Xiaoxia Wang, Caizhu Wang, Long Ma, Yanli Lyu, Han Liu, Fan Yang, Jiandong Wang, Longjiang Zhang, Jufang Wang

**Affiliations:** a School of Biology and Biological Engineering, South China University of Technology, Guangzhou, People's Republic of China; b Guangzhou Huayin Medical Laboratory Center Co, Ltd, Guangzhou, People's Republic of China; c Guangzhou Huayin Health Group Co., Ltd, Guangzhou, People's Republic of China; d Department of Pathology, Jinling Hospital, Affiliated Hospital of Medical School, Nanjing University, Nanjing, People's Republic of China; e Department of Radiology, Jinling Hospital, Affiliated Hospital of Medical School, Nanjing University, Nanjing, People's Republic of China; f School of Life Sciences, Jiangsu Key Laboratory for Molecular and Medical Biotechnology, Nanjing Normal University, Nanjing, People's Republic of China

**Keywords:** ARHGAP40, clear cell renal cell carcinoma, RhoA, PI3K/AKT signaling, glutamine-related metabolism, redox balance

## Abstract

**Objective:**

Clear cell renal cell carcinoma (ccRCC) is characterized by marked molecular and metabolic heterogeneity. This study investigated the functional role of ARHGAP40 in ccRCC and examined signaling and metabolic changes associated with ARHGAP40 expression.

**Methods:**

TCGA-KIRC and DiseaseMeth datasets were used to analyze ARHGAP40 expression and promoter methylation. Gain- and loss-of-function experiments evaluated malignant phenotypes *in vitro*, and an ARHGAP40-overexpression xenograft model was used to assess tumor growth. Co-immunoprecipitation and RhoA activity assays assessed ARHGAP40-RhoA interaction and RhoA activation, while PI3K/AKT signaling, glutamine metabolism, GSH/GSSG, and ROS levels were examined. Rescue experiments were performed using SC79, RhoA-Q63L, and NAC.

**Results:**

ARHGAP40 was downregulated in ccRCC and was associated with increased promoter methylation. ARHGAP40 overexpression suppressed cell proliferation, migration, invasion, EMT, and xenograft growth, while promoting apoptosis and G1-phase arrest; ARHGAP40 knockdown produced the opposite effects. Mechanistically, ARHGAP40 was associated with RhoA, and its overexpression was accompanied by reduced RhoA activity and PI3K/AKT phosphorylation. ARHGAP40 overexpression also reduced intracellular glutamine and glutamate levels and net glutamine consumption, decreased the GSH/GSSG ratio, and increased ROS levels. SC79 and RhoA-Q63L partially reversed the biological and redox effects induced by ARHGAP40 overexpression, whereas NAC reversed the redox changes.

**Conclusion:**

ARHGAP40 acts as a tumor suppressor in ccRCC. Our findings support a functional link between ARHGAP40, RhoA-PI3K/AKT signaling, glutamine-related metabolism, and cellular redox balance.

## Introduction

Renal cell carcinoma (RCC) is one of the most common malignancies of the urinary system, with a steadily increasing global incidence in recent years, imposing a growing clinical burden.^1^ Despite advances in imaging techniques and surgical interventions, a substantial proportion of patients are still diagnosed at advanced stages, when treatment options are limited and prognosis remains poor.^1^ Clear cell renal cell carcinoma (ccRCC) is the predominant histological subtype of RCC, accounting for approximately 70–75% of cases, and is characterized by aggressive behavior, a high propensity for metastasis, and pronounced molecular heterogeneity.^2^ ^,^ ^3^ Although targeted therapies and immune checkpoint inhibitors have improved survival outcomes in a subset of patients, therapeutic resistance remains common, and reliable biomarkers for assessing tumor aggressiveness and guiding individualized treatment are still lacking.^4^ Therefore, elucidating the molecular mechanisms underlying ccRCC progression and identifying key regulatory factors are of great importance for improving clinical management and patient prognosis.

Tumor invasion and metastasis are the primary causes of cancer-related mortality in ccRCC and depend on dynamic cytoskeletal remodeling and the acquisition of migratory and invasive capacities.^5^ Rho GTPases, including RhoA, Rac1, and Cdc42, are key molecular switches that link extracellular signals to actin cytoskeleton reorganization, thereby regulating cell shape, polarity, adhesion, and motility. Aberrant activation of Rho GTPase signaling has been implicated in tumor cell migration, epithelial-mesenchymal transition, and metastatic dissemination in multiple solid tumors, including RCC.^6^ The activity of Rho GTPases is tightly controlled by guanine nucleotide exchange factors (GEFs) and GTPase-activating proteins (GAPs), among which Rho GTPase-activating proteins (RhoGAPs) terminate Rho signaling and maintain cytoskeletal homeostasis.^7^ Increasing evidence suggests that dysfunction of RhoGAPs disrupts Rho signaling balance and confers sustained migratory and invasive advantages to tumor cells; however, the roles of specific RhoGAPs in ccRCC progression remain poorly defined.^8^

ARHGAP40 is an important member of the RhoGAP family and plays a role in regulating RhoA activity and actin cytoskeleton dynamics. Accumulating evidence indicates that ARHGAP40 functions as a potential tumor suppressor in multiple malignancies, and that epigenetic silencing mediated by promoter CpG island hypermethylation is a major mechanism underlying its downregulation.^9‐11^ Previous studies have demonstrated that ARHGAP40 is significantly downregulated in basal cell carcinoma and high-grade serous ovarian cancer due to promoter hypermethylation, and that loss of ARHGAP40 expression is closely associated with tumor progression and unfavorable clinical outcomes.^9^ ^,^ ^10^

Notably, our previous study in renal cell carcinoma primarily established the epigenetic and clinicopathological features of ARHGAP40: reduced ARHGAP40 expression was associated with promoter hypermethylation, tumor necrosis, and higher histological grade, and methylated ARHGAP40 DNA was detectable in circulating tumor DNA from patients with RCC.^12^ However, the biological effects of ARHGAP40 dysregulation and the signaling pathways involved were not investigated in that study.

In the present study, we extended our previous findings by investigating the effects of ARHGAP40 gain and loss in ccRCC cells and xenograft models. We further examined RhoA activity, PI3K/AKT signaling, glutamine-related metabolic changes, and redox balance. Rescue experiments using the AKT activator SC79, constitutively active RhoA-Q63L, and the antioxidant NAC were performed to evaluate the functional involvement of these signaling and redox changes. These findings extend our previous clinical and epigenetic observations by providing direct functional and mechanistic evidence for the role of ARHGAP40 in ccRCC.

## Materials and Methods

### Data Acquisition

ARHGAP40 expression data derived from The Cancer Genome Atlas (TCGA) and the Genotype-Tissue Expression (GTEx) project were downloaded through the HOME for Researchers platform (https://www.home-for-researchers.com/). The expression dataset comprised 532 TCGA-KIRC tumor samples and 161 normal kidney samples, including 72 from TCGA-KIRC and 89 from GTEx. The normal samples from both sources were combined into a single comparison group to evaluate differences in ARHGAP40 expression between tumor and normal tissues. Transcriptomic data were analyzed using gene annotation based on Homo_sapiens.GRCh38.gtf. Patients were divided into ARHGAP40-high and ARHGAP40-low groups using the median ARHGAP40 expression as the cutpoint. Differential expression was analyzed with limma using |log2FC| > 1 and Benjamini-Hochberg-adjusted *P* < 0.05.^13^ GO and KEGG enrichment analyzes were performed with clusterProfiler, and adjusted *P* < 0.05 was considered significant.^14^ GSEA (Gene Set Enrichment Analysis) was performed on ranked gene-expression data with 1,000 permutations, and FDR q < 0.05 was considered significant.^15^

DNA methylation data for ARHGAP40 were further analyzed using the DiseaseMeth (version 3.0), an integrative database that curates genome-wide DNA methylation profiles across human diseases, including 371 ccRCC samples and 206 normal kidney samples. Differential methylation patterns of ARHGAP40 between ccRCC and normal kidney tissues were evaluated.

### Cell lines and cell culture

Human ccRCC cell lines 786-O (CL-0010), A498 (CL-0254), and ACHN (CL-0021), as well as the human proximal tubule epithelial cell line HK-2 (CL-0109) and HEK 293 T (CL-0005) cells, were purchased from Procell (Wuhan, China). All cell lines were cultured in DMEM (11965092, Gibco, Shanghai, China) supplemented with 10% FBS (10099141 C, Gibco, Shanghai, China) and penicillin–streptomycin solution (P1400, Solarbio Life Sciences, Beijing, China), and maintained at 37 °C in a humidified incubator with 5% CO_2_. All cell lines were authenticated by short tandem repeat (STR) profiling and confirmed to be free of mycoplasma contamination before use.

### RNA interference and lentiviral system

Two independent siRNAs were used to silence ARHGAP40. siARHGAP40-1 (5′-CTCTGATTTGCTCAAAAGGTTCA-3′) and siARHGAP40-2 (sense, 5′-CUGCUAGAAGCUGACCACAAATT-3′; antisense, 5′-UUUGUGGUCAGCUUCUAGCAGTT-3′) were synthesized by Sangon Biotech (Shanghai, China). Corresponding non-targeting siRNA controls were used for each siRNA. siRNAs were transfected at a final concentration of 50 nM using Lipofectamine 2000 (Invitrogen; Cat. No. 11668-019). Cells were harvested or subjected to subsequent experiments 48 h after transfection.

For ARHGAP40 overexpression, ARHGAP40 was cloned into the pLVX-Puro vector. Lentiviral particles were generated in HEK293T cells by co-transfection of pLVX-Puro-ARHGAP40 or empty vector with psPAX2 and pMD2.G using Lipofectamine 2000. Viral supernatants were collected 48 h after transfection and used to infect target cells. Stable cells were selected with 2 μg/mL puromycin for 10 days and maintained in 0.5 μg/mL puromycin. ARHGAP40 overexpression was confirmed by RT-qPCR and western blotting before subsequent experiments.

### RT-qPCR

Total RNA was extracted using the RNApure Fast Tissue&Cell Kit (CW0599S; CWBIO, China), and 1 μg RNA was reverse-transcribed using M-MuLV Reverse Transcriptase (B600005; Sangon Biotech, China). RT-qPCR was performed with MagicSYBR Mixture (CW3008H; CWBIO) on an FQD-96A real-time PCR system (Bioer, China) in a 20-μL reaction system. Cycling conditions were 95 °C for 30 s, followed by 40 cycles of 95 °C for 5 s and 60 °C for 30 s. Each biological sample was analyzed in technical triplicate. GAPDH served as the reference gene, and relative expression was calculated using the 2^-ΔΔCt method. Reporting was expanded in accordance with the MIQE recommendations.^16^ ^,^ ^17^

Primer sequences were: ARHGAP40-F, 5′-AGCCTTCAACATGGACTCTGC-3′; ARHGAP40-R, 5′-TTTGGGGACGGTAACTTCGG-3′; GAPDH-F, 5′-GAAGGTGAAGGTCGGAGTCA-3′; and GAPDH-R, 5′-TTGAGGTCAATGAAGGGGTC-3’.

### Cell proliferation CCK8 assay

Cell proliferation was assessed using Cell Counting Kit-8 (CCK-8) assays (Cat. No. C0038, Beyotime Biotechnology, Shanghai, China). Cells were seeded in 96-well plates at 2,000 cells/well. Absorbance at 450 nm was recorded at 0, 24, 48, 72, and 96 h. At each measurement, 10 μL CCK-8 solution was added per well and incubated for 4 h before reading on a TECAN microplate reader (Männedorf, Switzerland). Experiments were independently repeated three times.

### Annexin V/PI apoptosis analysis

Apoptosis was assessed by Annexin V-FITC/propidium iodide (PI) staining using an Annexin V-FITC/PI apoptosis detection kit (Cat. No. KGA108, KeyGEN, Nanjing, China.). After the indicated treatments, 5 × 10^5^ cells were resuspended in binding buffer and incubated with 5 μL Annexin V-FITC and 5 μL PI for 15 min at room temperature in the dark. Samples were analyzed by flow cytometry (Beckman Colter, Fullerton, CA, USA). Debris was excluded by FSC/SSC gating and singlets were retained for analysis. At least 10,000 events were acquired per sample; unstained and single-stained controls were used to set gates and compensation. Annexin V−/PI− cells were considered viable, Annexin V+/PI− cells early apoptotic, and Annexin V+/PI+ cells late apoptotic. Data were analyzed using FlowJo.

### Cell cycle analysis

Cell-cycle distribution was analyzed by PI staining. Cells were harvested, washed twice with PBS, fixed in 70% ethanol, and stored at 4 °C until staining. Fixed cells were washed and incubated with 300 μL PI working solution (50 μg/mL in PBS; Cat. No. A601112, Sangon Biotech, Shanghai, China) and 50 μL RNase A working solution (Cat. No. R1030, Solarbio Science & Technology Co., Ltd., Beijing, China) at 37 °C for 1 h, followed by incubation at 4 °C overnight. Samples were analyzed using a CytoFLEX flow cytometer (Beckman Colter, Brea, CA, USA). Debris was excluded using FSC/SSC characteristics and doublets using FSC-A versus FSC-H gating. At least 10,000 singlet events were collected per sample, and G1, S, and G2/M fractions were quantified using FlowJo.

### Migration and invasion assay

For migration assays, 1.5 × 10^4^ cells in serum-free medium were seeded into uncoated 24-well Transwell inserts with 8-μm pores (Cat. No. 3422, Corning Inc., Corning, NY, USA), while 600 μL DMEM containing 20% FBS was added to the lower chamber. After 48 h, cells on the lower membrane surface were fixed with 4% formaldehyde for 20 min at 25 °C and stained with 0.1% crystal violet (Cat. No. G1064, Solarbio Life Sciences, Beijing, China) for 30 min. Three independent biological experiments were performed.

For invasion assays, Matrigel (Cat. No. 356234, Corning, Shanghai, China) was diluted 1:6 and 75 μL was applied to each Transwell insert. Cells were seeded and cultured under the same conditions as for migration assays. After 48 h, invaded cells were fixed and stained as described above. Cells were counted at × 100 magnification using a light microscope (Nikon Corporation, Tokyo, Japan).

### Xenograft model

The BALB/c-nu mice used in this study were obtained from Jiangsu Anokang Biotechnology Co., Ltd. (Jiangsu, China). Male BALB/c-nu mice aged 4-6 weeks and weighing approximately 18–22 g were used for the xenograft experiments. The animals were housed under specific pathogen-free conditions at Guangdong Laidi Biomedical Research Institute Co., Ltd., with a controlled temperature of 22 ± 2 °C, relative humidity of 50%–60%, and a 12-h light/dark cycle. Mice had free access to standard laboratory chow and sterile water and were allowed to acclimatize for one week before the experiment.

Control and ARHGAP40-overexpressing 786-O cells were used to establish subcutaneous xenografts in BALB/c-nu mice. Both the control and ARHGAP40 expression vectors contained a GFP reporter, allowing fluorescence-based monitoring of tumor growth in vivo. Twelve mice were allocated to the control and ARHGAP40-overexpression groups using a random-number method (*n* = 6 per group). Cells in exponential growth were harvested and resuspended at 1 × 10^6^ cells/100 μL PBS and injected subcutaneously into the flank. Tumor length and width were measured serially with vernier calipers by an investigator blinded to group allocation, and volume was calculated as 0.5 × length × width^2^. *In vivo* GFP fluorescence imaging was performed using an IVIS Lumina XR system with a 465-nm excitation filter and a GFP emission filter. The recorded acquisition settings were a 2-s exposure, binning of 8, an f-number of 2, and a 10-cm field of view.

All 12 enrolled mice completed the experiment and were analyzed for serial tumor volume, endpoint in vivo imaging, and tumor weight (*n* = 6 per group), with no exclusions. Serial caliper measurements were performed through day 20, as shown in Figure 3A, and mice were euthanized on day 21 post-implantation by CO2 inhalation followed by cervical dislocation; tumors were then excised and weighed. Animal reporting followed the ARRIVE 2.0 recommendations.^18^

All animal experiments were performed at Guangdong Laidi Biomedical Research Institute Co., Ltd. The experimental procedures were reviewed and approved by the Laboratory Animal Management and Ethics Committee of Guangdong Laidi Biomedical Research Institute Co., Ltd. (approval No. LDSW2025036-6). All animal procedures were conducted in accordance with institutional guidelines for the care and use of laboratory animals and complied with the ARRIVE guidelines.

### Co-immunoprecipitation assay

Co-immunoprecipitation (Co-IP) was performed to examine the association between ARHGAP40 and RhoA. For Flag-based Co-IP, cells expressing Flag-ARHGAP40 or the corresponding empty vector were lysed, and clarified lysates were subjected to immunoprecipitation using Magneti-G Anti-DYKDDDDK Beads (ACE Biotechnology, Changzhou, China; Cat. No. MP5101-01). After washing, the precipitated proteins were eluted and analyzed by western blotting for RhoA.

Endogenous Co-IP was further performed in 786-O and ACHN cells using an rProtein A/G Magnetic IP/Co-IP Kit (ACE Biotechnology, Changzhou, China; Cat. No. BK0004-01). Clarified cell lysates were incubated with mouse anti-RhoA monoclonal antibody (Invitrogen; Cat. No. MA1-134) or species-matched mouse IgG as a negative control. The immune complexes were captured with Protein A/G magnetic beads, washed, and subjected to western blotting with rabbit anti-ARHGAP40 antibody (MyBioSource; Cat. No. MBS9215052). Input lysates were included to confirm protein expression.

### RhoA activity assay

RhoA activity was measured using a configuration-specific RhoA Activation Assay Kit (Cat. No. 80601, NewEast Biosciences). Equal amounts of cell lysate were incubated with a conformation-specific anti-RhoA-GTP antibody to immunoprecipitate active GTP-bound RhoA, followed by collection with Protein A/G agarose beads and immunoblotting with anti-RhoA. Total RhoA was measured in whole-cell lysates. RhoA activity was expressed as the GTP-RhoA/total RhoA ratio. Three independent biological experiments were quantified.

### Western blotting

Western blotting was performed using SDS-PAGE and PVDF transfer. Membranes were blocked with 5% non-fat milk in TBST and incubated overnight at 4 °C with primary antibodies: *β*-actin (CST, 4970, 1:1000), ARHGAP40 (MyBioSource, MBS9215052, 1:1000), AKT (CST, 9272, 1:2000), phospho-AKT Ser473 (CST, 4060, 1:1000), PI3K p85 (CST, 4257, 1:1000), phospho-PI3K p85 Tyr458 (CST, 4228, 1:1000), RhoA (CST, 2117, 1:2000), E-cadherin (CST, 3195, 1:2000), *N*-cadherin (CST, 4061, 1:2000), and vimentin (CST, 5741, 1:2000). HRP-conjugated secondary antibody (CST, 7074, 1:2000) was used for detection with an enhanced chemiluminescence reagent (Beyotime Institute of Biotechnology, Shanghai, China). Densitometry was performed using Image Lab software (Bio-Rad Laboratories). Densitometric values were obtained from three independent biological experiments; phosphorylated proteins were normalized to their corresponding total proteins, GTP-RhoA to total RhoA, and other targets to *β*-actin.

### Intracellular reactive oxygen species (ROS) measurement by flow cytometry

Intracellular ROS was measured using a DCFH-DA-based Reactive Oxygen Species Assay Kit (Beyotime Institute of Biotechnology, Shanghai, China; Cat. No. S0033S). DCFH-DA was diluted 1:1000 in serum-free medium to a final concentration of 10 μM. Cells were incubated with the probe at 37 °C for 20 min in the dark, washed to remove excess probe, and analyzed by flow cytometry using 488-nm excitation and the FITC channel. Mean fluorescence intensity was quantified using FlowJo. Three independent biological experiments were performed.

### AKT activator (SC79) rescue experiment

For AKT rescue experiments, ARHGAP40-overexpressing and control 786-O and ACHN cells were treated with the AKT activator SC79 (S7863; Selleck Chemicals, Shanghai, China) at 5 μM, with an equal volume of dimethyl sulfoxide (DMSO) as vehicle control. Four groups were analyzed: oeCtrl+DMSO, oeARHGAP40+DMSO, oeCtrl+SC79, and oeARHGAP40+SC79. For single-time-point endpoints, cells were exposed to SC79 for 48 h before *p*-AKT/AKT western blotting, Annexin V/PI apoptosis analysis, ROS measurement, and GSH/GSSG determination. For proliferation experiments, cells were cultured in the presence of 5 μM SC79 and CCK-8 measurements were obtained at 0, 24, 48, 72, and 96 h. AKT activation was confirmed by an increased *p*-AKT/AKT ratio before functional interpretation.

### RhoA-Q63L rescue experiment

To test whether RhoA activity contributes upstream of AKT-associated effects, the constitutively active RhoA-Q63L coding sequence was cloned into the pLVX-Puro backbone, with empty pLVX-Puro used as the vector control, consistent with published pLVX-based RhoA-Q63L constructs. Control and ARHGAP40-overexpressing 786-O and ACHN cells were seeded in 6-well plates at approximately 2.5 × 10^5^ cells/well and transfected 24 h later using Lipofectamine 2000 (Invitrogen; Cat. No. 11668-019). For each well, 2.5 μg plasmid DNA was combined with 5 μL Lipofectamine 2000 (DNA:Lipofectamine 2000 ratio, 1 μg:2 μL), consistent with the manufacturer's 6-well format and published constitutively active Rho GTPase experiments. Four groups were generated: oeCtrl+Vector, oeARHGAP40+Vector, oeCtrl+RhoA-Q63L, and oeARHGAP40+RhoA-Q63L. Cells were analyzed 48 h after plasmid transfection. GTP-RhoA/total RhoA and *p*-AKT/AKT were measured to verify pathway rescue, followed by apoptosis, ROS, and GSH/GSSG assays. For CCK-8 experiments, cells were reseeded into 96-well plates after transfection and monitored from 0 to 96 h.

### *N*-acetylcysteine rescue experiment

For antioxidant rescue, control and ARHGAP40-overexpressing 786-O and ACHN cells were treated with *N*-acetyl-L-cysteine (NAC; ST1546-2g, ≥99%, reagent grade; Beyotime Institute of Biotechnology, Shanghai, China), generating oeCtrl, oeARHGAP40, oeCtrl+NAC, and oeARHGAP40+NAC groups. NAC was dissolved in sterile water and applied at a final concentration of 5 mM for 48 h before ROS and GSH/GSSG measurements.

### Measurement of glutamate and glutamine

Intracellular glutamate (Glu) and glutamine (Gln) were quantified using the Glutamic Acid Content Assay Kit (Boxbio, Beijing, China; Cat. No. AKAM002M) and Glutamine Content Assay Kit (Boxbio; Cat. No. AKAM027M), respectively, according to the manufacturer's protocols. Cells were harvested after the indicated treatments, lysed on ice, and clarified by centrifugation. Absorbance was measured at 450 nm on a TECAN microplate reader, and metabolite amounts were normalized to 10^6^ cells. Three independent biological experiments were performed.

### Extracellular glutamine consumption assay

To assess net extracellular glutamine consumption, 1 × 10^6^ cells were seeded in each well of a 6-well plate and cultured in 3 mL medium. Culture medium was collected at 0 and 24 h, and glutamine concentrations were measured using a Glutamine (Gln) Content Assay Kit (Boxbio, Beijing, China; Cat. No. AKAM027M). For each sample, 500 μL culture medium was mixed with 500 μL extraction solution B, vortexed for 5 min, and centrifuged at 12,000 × g for 5 min at 4 °C. The upper phase was collected, and absorbance was measured at 450 nm according to the manufacturer’s instructions. Glutamine concentration was calculated as 0.4 × ΔA_sample/ΔA_standard (μmol/mL). Net glutamine consumption over 24 h was calculated as (C0 − C24) × 3 mL and normalized to total cellular protein, with results expressed as μmol/mg protein/24 h.

### Measurement of reduced and oxidized glutathione

Reduced glutathione (GSH) and oxidized glutathione (GSSG) were measured using Boxbio kits (GSH, AKPR008M; GSSG, AKPR009M; Beijing, China) according to the manufacturer's instructions. Approximately 1 × 10^6^ cells were used per sample. Clarified cell lysates were used for the assays, GSSG was measured after the kit-specified derivatization/removal of GSH, and absorbance was read on a TECAN microplate reader. The GSH/GSSG ratio was calculated and normalized to cell number. Three independent biological experiments were performed.

### Statistical analysis

Statistical analyzes were performed using SPSS version 26.0 (IBM Corp., Armonk, NY, USA). For comparisons between two independent groups, two-tailed Student’s t-tests were used. Comparisons among multiple independent groups were performed using one-way ANOVA followed by Tukey’s multiple-comparisons test. For single-time-point rescue experiments involving two experimental factors, including SC79, RhoA-Q63L, and NAC treatments, two-way factorial ANOVA was used to evaluate the main effects and interaction between factors, followed by Tukey-adjusted multiple comparisons.​

For time-course experiments involving two groups, including conventional CCK-8 assays and serial xenograft tumor-volume measurements, two-way repeated-measures ANOVA was performed with experimental group as the between-subject factor and time as the repeated-measures factor. For CCK-8 rescue experiments involving ARHGAP40 status and either SC79 treatment or RhoA-Q63L expression, three-way mixed-design ANOVA was performed with ARHGAP40 status and rescue treatment as between-subject factors and time as the repeated-measures factor. Geisser–Greenhouse correction was applied to effects involving time, followed by prespecified Šídák-adjusted multiple comparisons where appropriate.​

All in vitro experiments were independently repeated three times (biological *n* = 3), unless otherwise specified, and animal experiments included six mice per group. Data are presented as mean ± SEM. Adjusted *P* < 0.05 was considered statistically significant.

## Results

### ARHGAP40 is downregulated and hypermethylated in ccRCC

Analysis of the integrated TCGA-KIRC and GTEx datasets showed that ARHGAP40 expression was markedly lower in ccRCC tissues (*n* = 532) than in normal kidney tissues (*n* = 161; *P* < 0.0001, Figure 1A). Consistent with the corrected methylation analysis, DiseaseMeth data showed significantly higher ARHGAP40 methylation in ccRCC tissues (*n* = 371) than in normal kidney tissues (*n* = 206; *P* < 0.001, Figure 1B). ARHGAP40 mRNA and protein expression were also reduced in the ccRCC cell lines A498, 786-O, and ACHN relative to HK-2 cells (Figure 1C,D). Among the tumor cell lines, 786-O and ACHN exhibited relatively low endogenous ARHGAP40 expression, whereas A498 cells retained comparatively higher expression, supporting their use for overexpression and knockdown experiments, respectively.

**Figure 1.**
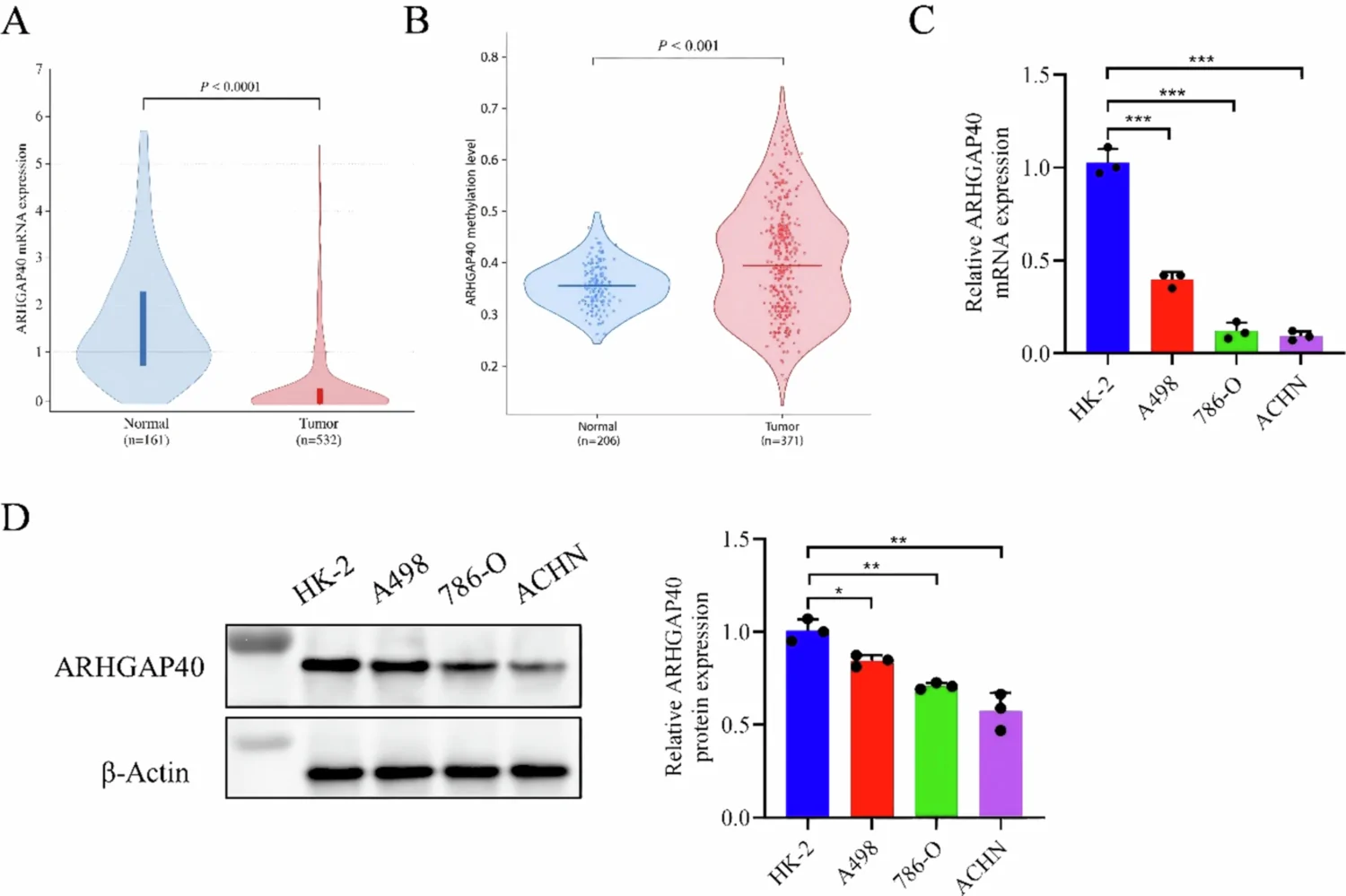
ARHGAP40 is downregulated and hypermethylated in ccRCC. (A) ARHGAP40 mRNA expression in TCGA-KIRC tumor tissues (*n* = 532) and normal kidney tissues from TCGA-KIRC (*n* = 72) and GTEx (*n* = 89). Expression data were obtained through the HOME for Researchers platform. (B) ARHGAP40 methylation levels in normal kidney tissues (*n* = 206) and ccRCC tissues (*n* = 371) from DiseaseMeth (version 3.0). (C) Relative ARHGAP40 mRNA expression in HK-2, A498, 786-O, and ACHN cells determined by RT-qPCR. (D) Representative western blot and densitometric analysis of ARHGAP40 protein expression in the indicated cell lines. For cell-line experiments, *n* = 3 independent biological replicates and data are presented as mean ± SEM. Cell-line comparisons were analyzed by one-way ANOVA followed by Tukey's multiple-comparisons test; database *P* values are shown in the corresponding panels. * *P* < 0.05, ** *P* < 0.01, *** *P* < 0.001.

### ARHGAP40 suppresses ccRCC cell growth, migration, invasion, and EMT

ARHGAP40 overexpression in 786-O and ACHN cells and knockdown with siARHGAP40-1 in A498 cells were confirmed by RT-qPCR (Supplementary Fig. S1A, S2A). ARHGAP40 overexpression significantly reduced cell proliferation and increased the proportion of Annexin V-positive cells, whereas ARHGAP40 knockdown produced the opposite effects (Figure 2A,B, Supplementary Fig. S2B, C). Cell-cycle analysis further showed that ARHGAP40 overexpression shifted cells toward G1-phase arrest, while ARHGAP40 depletion promoted cell-cycle progression (Supplementary Fig. S1B, C). In parallel, ARHGAP40 overexpression suppressed Transwell migration and invasion in 786-O and ACHN cells, whereas ARHGAP40 knockdown enhanced both phenotypes in A498 cells (Figure 2C,D). Western blotting showed increased E-cadherin and decreased *N*-cadherin and vimentin after ARHGAP40 overexpression, with reciprocal changes after ARHGAP40 knockdown (Figure 2E), indicating that ARHGAP40 restrains EMT-associated phenotypic changes in ccRCC cells.

**Figure 2.**
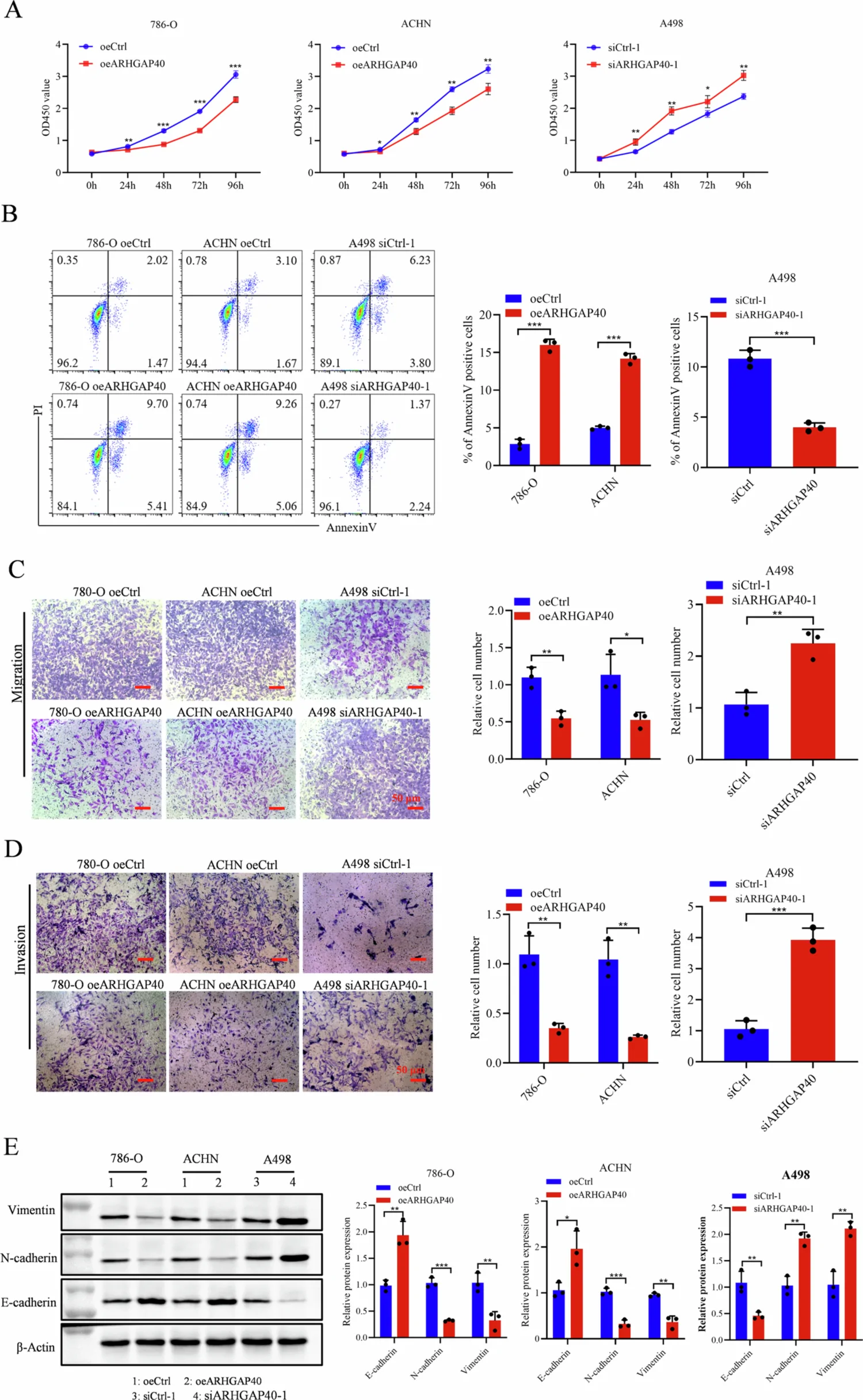
ARHGAP40 suppresses proliferation, migration, invasion, and epithelial-mesenchymal transition (EMT)-associated changes in ccRCC cells. (A) CCK-8 proliferation curves for 786-O and ACHN cells with ARHGAP40 overexpression and A498 cells transfected with siARHGAP40-1. (B) Representative Annexin V/PI flow-cytometry plots and quantification of Annexin V-positive cells. (C) Representative Transwell migration images and quantification. Scale bar, 50 μm. (D) Representative Transwell invasion images and quantification. Scale bar, 50 μm. (E) Representative western blots and densitometric analyzes of E-cadherin, *N*-cadherin, and vimentin. *n* = 3 independent biological replicates; data are presented as mean ± SEM. Time-course CCK-8 data were analyzed by two-way repeated-measures ANOVA with Geisser-Greenhouse correction, followed by Šídák-adjusted multiple comparisons between groups at each time point. For 786-O cells, significant effects were observed for ARHGAP40 status (*P* < 0.0001), time (*P* < 0.0001), and the ARHGAP40 × time interaction (*P* = 0.00011). For ACHN cells, the corresponding *P* values were < 0.0001, < 0.0001, and 0.0043, respectively; for A498 cells, they were 0.0037, < 0.0001, and 0.0012, respectively. Other two-group comparisons were analyzed using two-tailed Student's t-tests. * *P* < 0.05, ** *P* < 0.01, *** *P* < 0.001.

### ARHGAP40 overexpression suppresses ccRCC xenograft growth in vivo

The tumor-suppressive effect of ARHGAP40 was further evaluated in a 786-O xenograft model, with six mice included in each group and no exclusions. Tumors derived from ARHGAP40-overexpressing cells grew more slowly than control tumors across the observation period (Figure 3A). In vivo imaging at the experimental endpoint showed a lower fluorescence signal in the ARHGAP40-overexpression group (Figure 3B). Excised tumors were visibly smaller (Figure 3C), and final tumor weight was significantly reduced compared with the control group (Figure 3D). These data confirm that ARHGAP40 overexpression suppresses ccRCC tumor growth in vivo.

**Figure 3.**
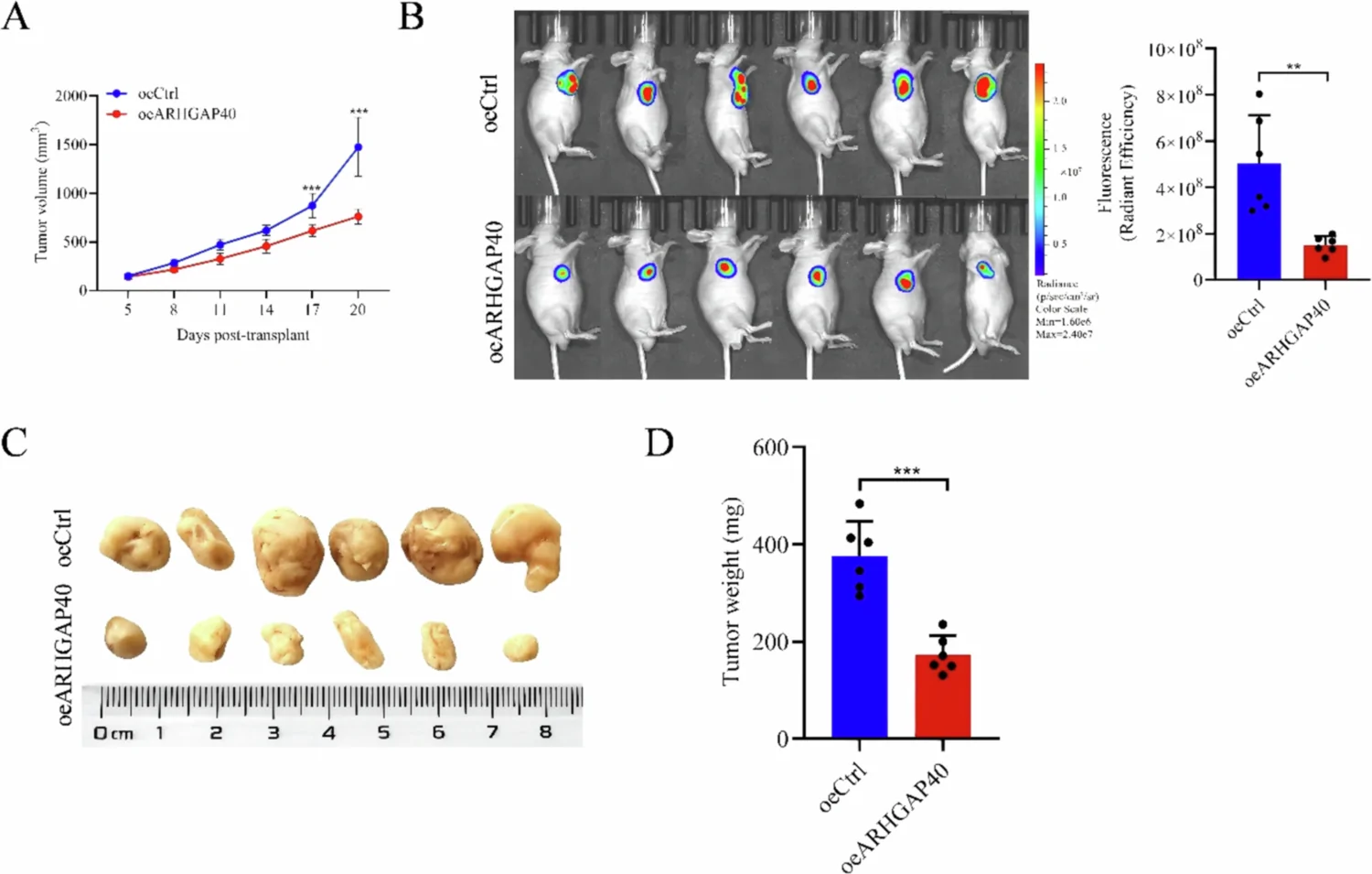
ARHGAP40 overexpression suppresses ccRCC xenograft growth in vivo. (A) Tumor-volume curves for 786-O oeCtrl and oeARHGAP40 xenografts. (B) Representative in vivo optical images and quantitative radiant-efficiency measurements at the endpoint. (C) Excised xenograft tumors at the endpoint. (D) Final tumor weights. Six mice were enrolled and analyzed per group (*n* = 6), with no exclusions. Data are presented as mean ± SEM. Data are presented as mean ± SEM. Serial tumor-volume measurements were analyzed using two-way repeated-measures ANOVA, with group as the between-subject factor and time as the within-subject factor. The Geisser–Greenhouse correction was applied to effects involving time. Between-group comparisons at the six time points were adjusted using the Šídák method. Significant effects were observed for group (F(1, 10) = 89.03, *P* < 0.0001), time (F(1.52,15.20) = 134.61, *P* < 0.0001), and the group × time interaction (F(1.52,15.20) = 17.11, *P* = 0.00028). Adjusted between-group differences were significant on days 17 and 20, but not on days 5, 8, 11, or 14.** *P* < 0.01, *** *P* < 0.001.

### Bioinformatic analyzes implicate RhoA-associated signaling and metabolic pathways in ARHGAP40-related effects

To identify molecular processes associated with ARHGAP40, STRING analysis was used to construct an ARHGAP40-centered protein-association network, in which RhoA was among the linked proteins (Figure 4A). Gene Ontology analysis of the STRING-associated proteins showed enrichment in cell-cell junction, cell junction, dendrite/axon, synapse, and related cellular-component terms (Figure 4B). AlphaFold3 modeling generated a putative ARHGAP40-RhoA complex with an ipTM score of 0.74 and a pTM score of 0.61 (Figure 4C). In the TCGA-KIRC cohort, KEGG analysis of differentially expressed genes between ARHGAP40-high and ARHGAP40-low tumors identified enrichment of PI3K-AKT signaling, PPAR signaling, ECM-receptor interaction, and alanine, aspartate and glutamate metabolism, among other pathways (Figure 4D). Additional GO analysis highlighted organic anion/carboxylic acid transport, transporter activity, and xenobiotic-response terms, whereas GSEA showed significant negative enrichment of multiple signaling and metabolic gene sets (FDR q < 0.05; Supplementary Fig. S3A, B).

**Figure 4.**
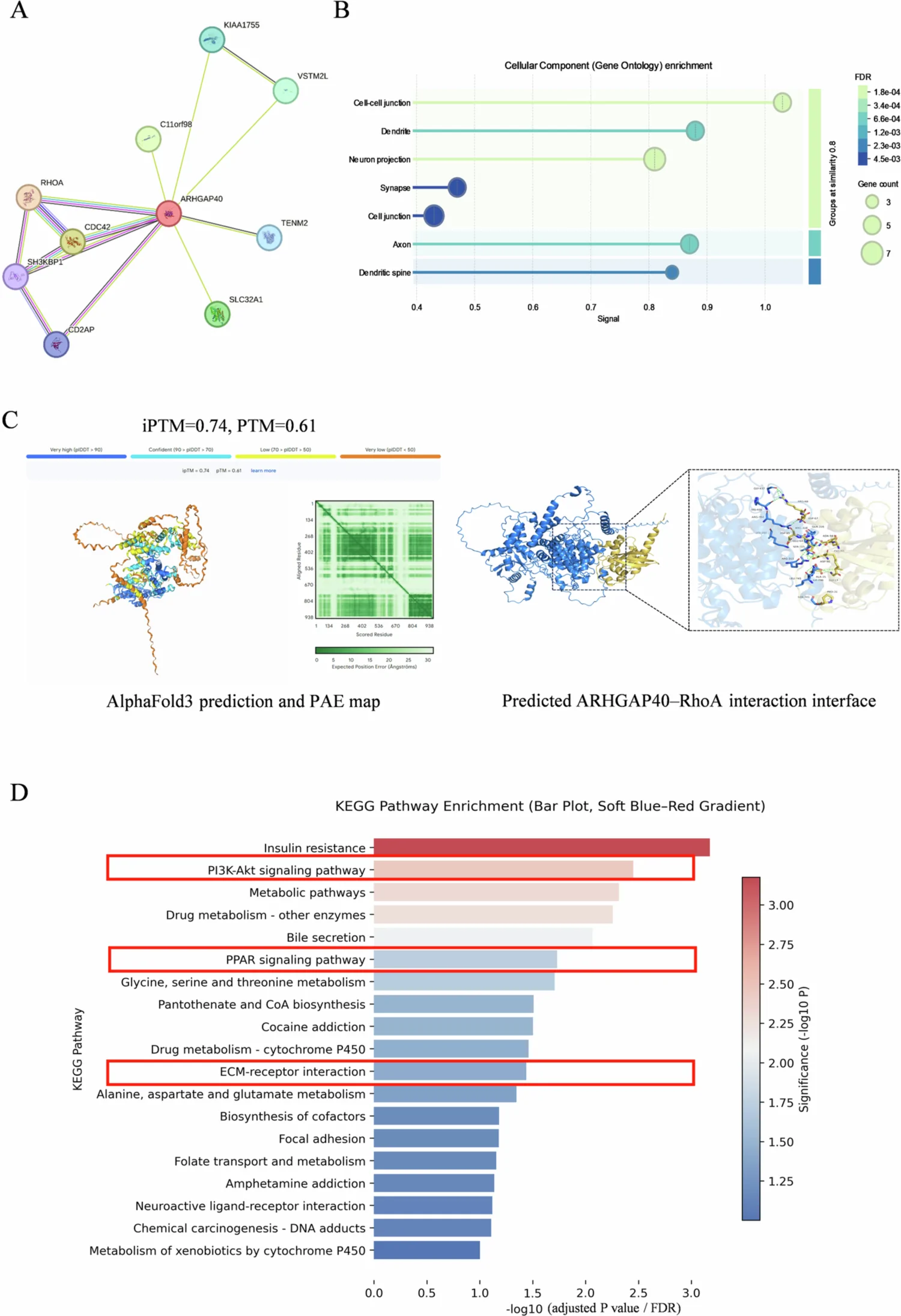
Bioinformatic and structural analyzes identify RhoA-associated signaling and metabolic pathways related to ARHGAP40. (A) STRING protein-association network centered on ARHGAP40. (B) Gene Ontology cellular-component enrichment of STRING-associated proteins; color denotes false discovery rate (FDR) and point size denotes gene count. (C) AlphaFold3 prediction of the putative ARHGAP40-RhoA complex, including the predicted aligned error (PAE) map, interface predicted template modeling score (ipTM = 0.74), predicted template modeling score (pTM = 0.61), and modeled interface. (D) KEGG pathway enrichment of differentially expressed genes between ARHGAP40-high and ARHGAP40-low TCGA-KIRC tumors. The TCGA-KIRC tumor cohort contained *n* = 532 samples and was divided at the median ARHGAP40 expression level. Differential-expression and enrichment *P* values were adjusted using the Benjamini-Hochberg procedure; adjusted *P* < 0.05 was considered significant.

### ARHGAP40 is associated with reduced RhoA activation and PI3K/AKT signaling and with altered glutamine-related and redox measures

Co-immunoprecipitation experiments showed association of ARHGAP40 with RhoA in ccRCC cells. This was observed in Flag-based immunoprecipitation and was further supported by endogenous immunoprecipitation of RhoA in 786-O and ACHN cells (Figure 5A,B). Consistent with the RhoA activity assay used in the Methods, immunoprecipitation of GTP-bound RhoA with a conformation-specific anti-RhoA-GTP antibody showed that ARHGAP40 overexpression decreased the GTP-RhoA/total RhoA ratio, whereas ARHGAP40 knockdown increased it (Figure 5C). ARHGAP40 overexpression was also accompanied by reduced *p*-PI3K/PI3K and *p*-AKT/AKT ratios, while ARHGAP40 silencing increased both phosphorylation ratios (Figure 5D). At the metabolic level, ARHGAP40 overexpression reduced intracellular glutamate and glutamine pools in 786-O and ACHN cells, whereas ARHGAP40 knockdown increased both metabolites in A498 cells (Figure 5E,F). Measurement of extracellular glutamine consumption over 24 h showed a corresponding decrease after ARHGAP40 overexpression and an increase after ARHGAP40 knockdown (Supplementary Fig. S4A, B). ARHGAP40 overexpression also decreased the GSH/GSSG ratio and increased intracellular ROS, with reciprocal changes after ARHGAP40 knockdown (Figure 5G,H). Together, these data link ARHGAP40 expression to RhoA activity, PI3K/AKT signaling, glutamine-related metabolite availability, glutamine consumption, and cellular redox balance without inferring unmeasured metabolic flux or glutathione synthesis rates.

**Figure 5.**
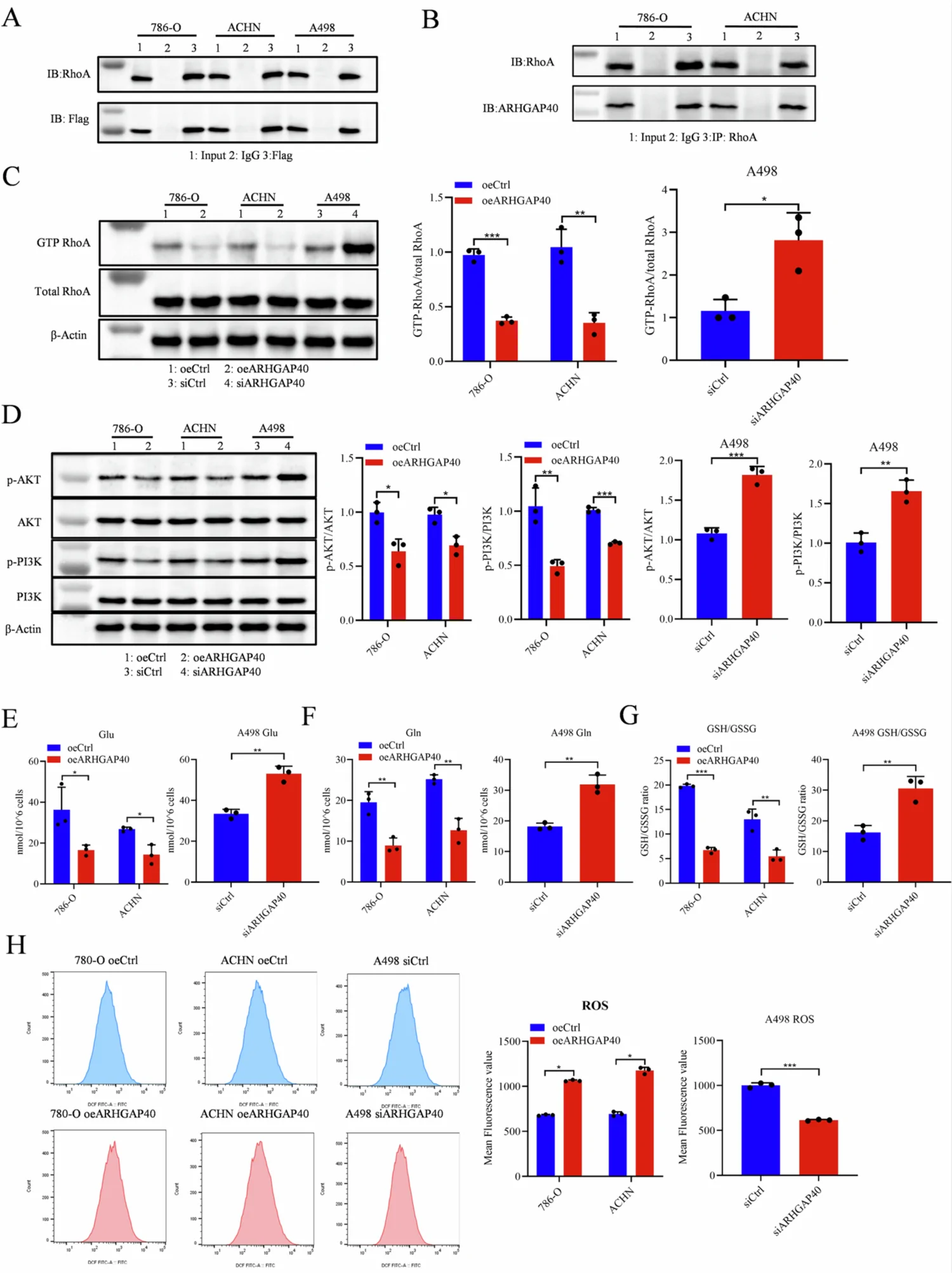
ARHGAP40 associates with RhoA and is accompanied by reduced RhoA activity, PI3K/AKT signaling, and altered glutamine-related/redox measures. (A) Flag-based co-immunoprecipitation showing association of ARHGAP40 with RhoA. (B) Endogenous immunoprecipitation of RhoA followed by immunoblotting for ARHGAP40. (C) RhoA activity assay using a conformation-specific anti-RhoA-GTP antibody, with representative GTP-RhoA and total RhoA blots and quantification of the GTP-RhoA/total RhoA ratio. (D) Representative western blots and densitometric analyzes of *p*-AKT/AKT and *p*-PI3K/PI3K. (E) Intracellular glutamate levels. (F) Intracellular glutamine levels. (G) GSH/GSSG ratios. (H) Representative ROS flow-cytometry histograms and mean fluorescence intensity. Quantitative analyzes used *n* = 3 independent biological replicates and are shown as mean ± SEM. Two-group comparisons were performed using two-tailed Student's t-test. * *P* < 0.05, ** *P* < 0.01, *** *P* < 0.001.

### AKT activation partially reverses ARHGAP40-associated growth and redox phenotypes

To test whether reduced AKT activity contributes to the ARHGAP40-associated phenotype, ARHGAP40-overexpressing 786-O and ACHN cells were treated with the AKT activator SC79. SC79 increased the *p*-AKT/AKT ratio in both control and ARHGAP40-overexpressing cells, confirming pharmacological AKT activation (Figure 6A). Relative to ARHGAP40 overexpression with vehicle, SC79 partially restored cell proliferation and markedly reduced the increase in apoptosis (Figure 6B,C). SC79 also decreased ARHGAP40-associated ROS accumulation and partially restored the GSH/GSSG ratio (Figure 6D,E). These rescue results support AKT signaling as a functional contributor to both the growth and redox-associated effects observed after ARHGAP40 overexpression.

**Figure 6.**
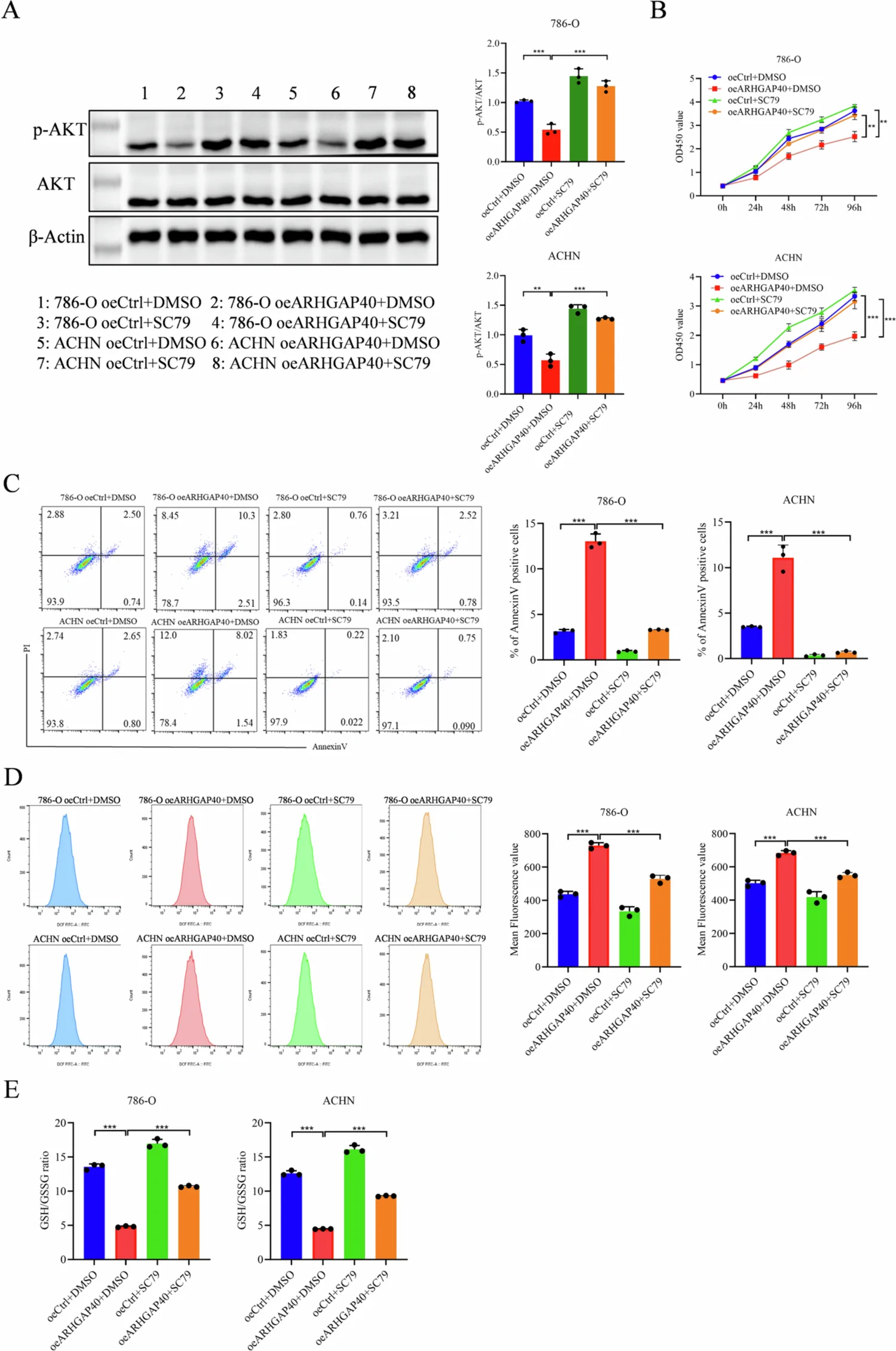
AKT activation with SC79 partially rescues ARHGAP40-associated growth and redox phenotypes. (A) Representative *p*-AKT, AKT, and *β*-actin western blots and *p*-AKT/AKT quantification. (B) CCK-8 proliferation curves. (C) Annexin V/PI flow cytometry and quantification. (D) ROS histograms and mean fluorescence intensity. (E) GSH/GSSG ratios. *n* = 3 independent biological replicates; data are presented as mean ± SEM. Single-time-point panels were analyzed by two-way factorial ANOVA (ARHGAP40 status × SC79 treatment) followed by Tukey-adjusted multiple comparisons. CCK-8 data were analyzed by a three-way mixed-design ANOVA with ARHGAP40 status and SC79 treatment as factorial variables and time as the repeated-measures factor; Geisser-Greenhouse correction was applied to effects involving time, followed by prespecified Šídák-adjusted multiple comparisons. In 786-O cells, the ARHGAP40 × SC79 interaction was significant (F(1, 8) = 15.69, *P* = 0.0042), as was the ARHGAP40 × SC79 × time interaction (F(1.89,15.15) = 4.17, *P* = 0.0378). In ACHN cells, the corresponding interactions were also significant (F(1, 8) = 15.07, *P* = 0.0047; and F(2.48,19.87) = 10.62, *P* = 0.00039, respectively). ** *P* < 0.01, *** *P* < 0.001.

### Constitutively active RhoA partially restores AKT signaling and reverses ARHGAP40-associated cellular phenotypes

To further evaluate pathway order, the constitutively active RhoA mutant RhoA-Q63L was introduced into 786-O and ACHN cells. RhoA-Q63L increased the GTP-RhoA/total RhoA ratio and partially restored AKT phosphorylation in ARHGAP40-overexpressing cells (Figure 7A–C). Functionally, RhoA-Q63L partially rescued the reduction in cell proliferation and attenuated ARHGAP40-induced apoptosis (Figure 7D,E). RhoA-Q63L also reduced ROS accumulation and partially restored the GSH/GSSG ratio in ARHGAP40-overexpressing cells (Figure 7F,G). These data support RhoA activity as an upstream contributor to the AKT and redox-associated changes observed after ARHGAP40 overexpression, while not excluding additional parallel signaling inputs.

**Figure 7.**
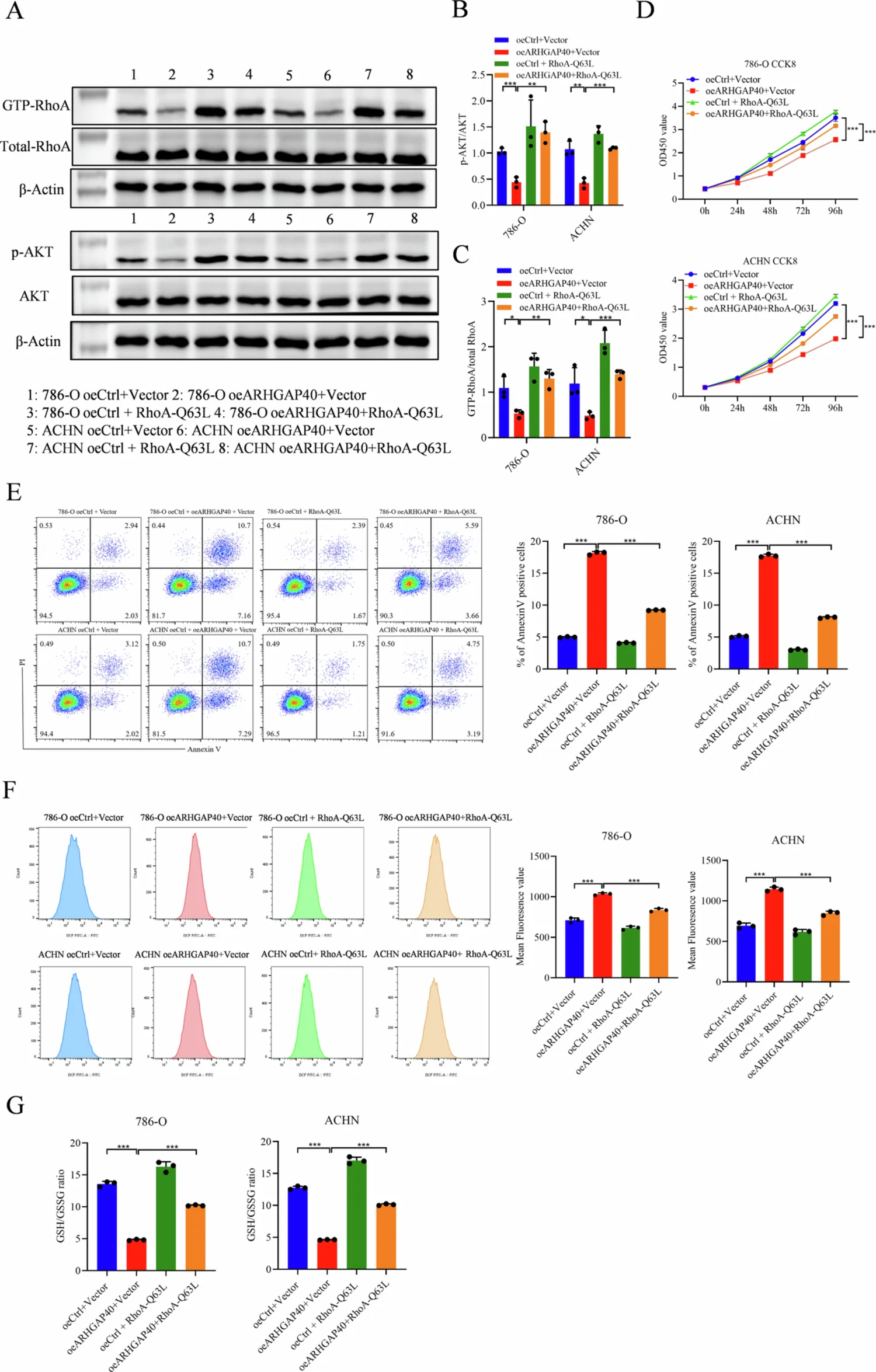
Constitutively active RhoA partially restores AKT signaling and reverses ARHGAP40-associated growth and redox phenotypes. (A) Representative GTP-RhoA, total RhoA, *p*-AKT, AKT, and *β*-actin western blots. (B) *p*-AKT/AKT ratios. (C) GTP-RhoA/total RhoA ratios. (D) CCK-8 proliferation curves obtained after reseeding transfected cells. (E) Annexin V/PI flow cytometry and quantification. (F) ROS histograms and mean fluorescence intensity. (G) GSH/GSSG ratios. *n* = 3 independent biological replicates; data are presented as mean ± SEM. Single-time-point panels were analyzed by two-way factorial ANOVA (ARHGAP40 status × RhoA-Q63L expression), followed by Tukey-adjusted multiple comparisons. CCK-8 data were analyzed by a three-way mixed-design ANOVA with ARHGAP40 status and RhoA-Q63L expression as between-subject factors and time as the repeated-measures factor. Geisser-Greenhouse correction was applied to effects involving time, followed by prespecified Šídák-adjusted multiple comparisons. In 786-O cells, the ARHGAP40 × RhoA-Q63L interaction was significant (F(1, 8) = 27.22, *P* = 0.00081), whereas the ARHGAP40 × RhoA-Q63L × time interaction did not reach statistical significance after Geisser-Greenhouse correction (F(2.18,17.41) = 3.39, *P* = 0.0536). In ACHN cells, both the ARHGAP40 × RhoA-Q63L interaction (F(1, 8) = 26.35, *P* = 0.00089) and the ARHGAP40 × RhoA-Q63L × time interaction (F(1.01,8.09) = 72.82, *P* < 0.0001) were significant. * *P* < 0.05, ** *P* < 0.01, *** *P* < 0.001.

### *N*-acetylcysteine reverses ARHGAP40-associated redox changes

Finally, antioxidant rescue experiments were performed using *N*-acetylcysteine (NAC). NAC markedly reduced the increase in intracellular ROS caused by ARHGAP40 overexpression in 786-O and ACHN cells and partially restored the GSH/GSSG ratio (Supplementary Fig. S5A, B). Thus, the redox alterations associated with ARHGAP40 overexpression are reversible by antioxidant treatment.

## Discussion

Renal cell carcinoma, particularly clear cell renal cell carcinoma, is characterized by marked molecular complexity and adaptive capacity. Its development and progression depend not only on sustained activation of canonical oncogenic signaling pathways but also on extensive metabolic reprogramming and dynamic regulation of redox homeostasis.^19^ Originating from proximal tubular epithelial cells, ccRCC displays distinctive features, including abnormal lipid and glycogen accumulation, enhanced tolerance to oxidative stress, and pronounced signaling plasticity.^20^ These interrelated metabolic and signaling adaptations collectively contribute to tumor aggressiveness and influence the heterogeneous responses of ccRCC to targeted and immune-based therapies.

The Rho GTPase family has emerged as a key regulatory hub linking cytoskeletal remodeling to intracellular signaling during tumor progression. While RhoA has traditionally been regarded as a regulator of actin cytoskeleton dynamics and cell motility, increasing evidence indicates that RhoA signaling also intersects with major oncogenic pathways, including PI3K/AKT, thereby influencing tumor cell proliferation, survival, and stress adaptation.^21^ ^,^ ^22^ In ccRCC, aberrant activation of RhoA signaling has been associated with increased invasiveness and adverse clinical outcomes; however, the upstream mechanisms restraining RhoA activity and their relationship to metabolic reprogramming remain incompletely understood.^21^ ^,^ ^23^

ARHGAP40 is a member of the Rho GTPase-activating protein (RhoGAP) family. Our earlier RCC study established promoter hypermethylation, reduced ARHGAP40 expression, and their clinicopathological associations, but did not test whether ARHGAP40 actively regulates ccRCC phenotypes or define its signaling context. The present study therefore advances that work by demonstrating gain- and loss-of-function effects on proliferation, apoptosis, cell-cycle progression, migration, invasion, EMT-associated markers, and xenograft growth, and by examining RhoA/PI3K-AKT and metabolic-redox changes associated with ARHGAP40 manipulation.

The expression and methylation findings in the current dataset are consistent with our prior clinical observations.^12^ Extending these observations, functional analyzes further support a tumor-suppressive role for ARHGAP40 in ccRCC. Both *in vitro* and *in vivo* experiments consistently show that enforced ARHGAP40 expression inhibits RCC cell proliferation, induces apoptosis and cell-cycle arrest, and suppresses migration and invasion, accompanied by partial reversal of epithelial-mesenchymal transition-associated molecular changes. These findings are consistent with the established role of Rho GTPase signaling in cytoskeletal regulation and tumor invasiveness and further support its involvement in broader control of tumor cell state.

Mechanistically, the present data support an association between ARHGAP40 and RhoA rather than proving direct binding. STRING and AlphaFold3 analyzes generated a plausible interaction model, while Flag-based and endogenous Co-IP demonstrated protein association in cells. The conformation-specific RhoA activity assay showed reduced GTP-RhoA after ARHGAP40 overexpression and increased GTP-RhoA after ARHGAP40 knockdown. In addition, constitutively active RhoA-Q63L partially restored AKT phosphorylation, proliferation, apoptosis, and redox readouts in ARHGAP40-overexpressing cells, providing functional pathway-order evidence. This interpretation is consistent with mechanistic work showing that RhoGAP activity can be strongly shaped by domain interactions and that mutant/rescue approaches are useful for distinguishing association from GAP-dependent regulation.^24^ ^,^ ^25^

The relationship between RhoA and PI3K/AKT signaling is unlikely to be a strictly isolated linear cascade. RhoA can engage adhesion- and cytoskeleton-associated signaling, including ROCK- and FAK-related pathways, which can intersect with PI3K/AKT signaling.^22^ ^,^ ^26^ In our experiments, ARHGAP40 overexpression reduced *p*-PI3K/PI3K and *p*-AKT/AKT, SC79 partially reversed the growth and redox phenotypes, and RhoA-Q63L partially restored AKT phosphorylation. Together, these results support a functional RhoA-to-AKT contribution while leaving open the possibility of parallel inputs and feedback regulation.

Recent studies have shown that the PIK3CA E545K mutation can activate AKT signaling and alter sensitivity to AKT inhibition,^27^ while distinct activating AKT mutations differ in their signaling effects and responses to AKT inhibitors.^28^ These findings highlight the importance of pathway genetic background when interpreting the effects of experimental interventions. However, the mutation status of genes involved in this pathway was not systematically assessed in the cell lines or patient datasets analyzed in the present study. Such genetic differences could influence basal signaling activity and responses to ARHGAP40 manipulation or rescue interventions, potentially contributing to variability among experimental models. Future studies should characterize the relevant mutation profiles and compare ARHGAP40-associated effects across models with distinct PI3K/AKT pathway genetic backgrounds.

The metabolic changes observed in this study further indicate that ARHGAP40 may be involved in the regulation of glutamine-related metabolic processes and redox balance in ccRCC. ARHGAP40 overexpression reduced intracellular glutamine and glutamate pools and decreased net glutamine consumption from the culture medium over 24 h, whereas ARHGAP40 knockdown produced reciprocal changes. ARHGAP40 overexpression also lowered the GSH/GSSG ratio and increased ROS. These observations are consistent with altered glutamine-related metabolism and antioxidant buffering in ccRCC,^29‐31^ but they do not by themselves define isotope-resolved glutamine flux, a specific transporter, or a glutathione synthesis rate. The revised title and conclusions therefore refer to glutamine-related metabolic measures and redox balance rather than glutamine dependence or a fully established metabolic flux mechanism.

The rescue experiments provide further evidence that the signaling changes associated with ARHGAP40 are functionally linked to downstream redox regulation. SC79 and RhoA-Q63L both partially restored the GSH/GSSG ratio and reduced ROS accumulation in ARHGAP40-overexpressing cells, linking the signaling changes to downstream redox phenotypes. NAC also reversed the ROS and GSH/GSSG changes, demonstrating that these redox readouts are antioxidant-sensitive.

This study has several limitations. First, direct binding between ARHGAP40 and RhoA and the dependence of this regulation on ARHGAP40 GAP activity remain to be further validated using purified proteins or functional mutants. Second, PI3K/AKT pathway mutation status was not assessed. Whether it affects the response to ARHGAP40 manipulation warrants investigation in future studies. Third, isotope-tracing studies were not performed, and the specific glutamine transporters or metabolic enzymes involved were not identified. In addition, the NAC experiments only demonstrated reversal of redox changes and did not establish a direct causal role for ROS in ARHGAP40-mediated growth suppression. Finally, the related signaling and redox alterations were not further validated in xenograft tissues.

## Conclusions

In summary, ARHGAP40 exerts tumor-suppressive effects in ccRCC and is associated with reduced RhoA/PI3K-AKT signaling, altered glutamine-related metabolism, and redox imbalance. These findings identify ARHGAP40 as a potential regulator of ccRCC progression and provide a basis for further investigation of its signaling and metabolic mechanisms.

## Supplementary Material

Supplementary Figure 1_.docx Supplementary Figure 1_.docx

Supplementary Figure 5_.docx Supplementary Figure 5_.docx

Supplementary Figure 3_.docx Supplementary Figure 3_.docx

Supplementary Figure 2_.docx Supplementary Figure 2_.docx

Supplementary Figure S4.jpg

Supplementary captions.docx Supplementary captions.docx

ARRIVE_Checklist_20260930.pdf ARRIVE_Checklist_20260930.pdf

## Data Availability

The data supporting the findings of this study are available in Figshare at https://doi.org/10.6084/m9.figshare.32647410.
