# Supplementary material for: ARHGAP40 suppresses clear cell renal cell carcinoma progression and is associated with altered glutamine-related metabolism and redox balance
Source: Cancer Biol Ther. 2026 Oct 8;27(1):2746137. doi: 10.1080/15384047.2026.2746137 (PMC13652080; doi:10.1080/15384047.2026.2746137)
Supplement: Supplementary file 1 [file KCBT_A_2746137_SM3151.pdf]

The ARRIVE guidelines 2.0: author checklist

The ARRIVE Essential 10

These items are the basic minimum to include in a manuscript. Without this information, readers and reviewers cannot assess the reliability of the findings.

Item		Recommendation	Section/line number, or reason for not reporting
**Study design**	1	For each experiment, provide brief details of study design including:	P. 7, lines 32-34; P. 8, lines 1-4.
		a. The groups being compared, including control groups. If no control group has been used, the rationale should be stated.	
		b. The experimental unit (e.g. a single animal, litter, or cage of animals).	P. 8, lines 1-4, 10-12.
**Sample size**	2	a. Specify the exact number of experimental units allocated to each group, and the total number in each experiment. Also indicate the total number of animals used.	P. 8, lines 1-2, 10-12.
		b. Explain how the sample size was decided. Provide details of any *a priori* sample size calculation, if done.	Not reported.
**Inclusion and exclusion criteria**	3	a. Describe any criteria used for including and excluding animals (or experimental units) during the experiment, and data points during the analysis. Specify if these criteria were established *a priori*. If no criteria were set, state this explicitly.	Not reported.
		b. For each experimental group, report any animals, experimental units or data points not included in the analysis and explain why. If there were no exclusions, state so.	P. 8, lines 10-12.
		c. For each analysis, report the exact value of *n* in each experimental group.	P. 8, lines 10-12; P. 23, lines 9-10.
**Randomisation**	4	a. State whether randomisation was used to allocate experimental units to control and treatment groups. If done, provide the method used to generate the randomisation sequence.	P. 8, lines 1-2.
		b. Describe the strategy used to minimise potential confounders such as the order of treatments and measurements, or animal/cage location. If confounders were not controlled, state this explicitly.	Not reported.
**Blinding**	5	Describe who was aware of the group allocation at the different stages of the experiment (during the allocation, the conduct of the experiment, the outcome assessment, and the data analysis).	P. 8, lines 4-5.
**Outcome measures**	6	a. Clearly define all outcome measures assessed (e.g. cell death, molecular markers, or behavioural changes).	P. 8, lines 4-14; P. 23, lines 5-10.
		b. For hypothesis-testing studies, specify the primary outcome measure, i.e. the outcome measure that was used to determine the sample size.	Not reported.
**Statistical methods**	7	a. Provide details of the statistical methods used for each analysis, including software used.	P. 11, lines 29-34; P. 12, lines 1-15; P. 23, lines 11-20.
		b. Describe any methods used to assess whether the data met the assumptions of the statistical approach, and what was done if the assumptions were not met.	P. 12, lines 9-11; P. 23, lines 14-15.
**Experimental animals**	8	a. Provide species-appropriate details of the animals used, including species, strain and substrain, sex, age or developmental stage, and, if relevant, weight.	P. 7, lines 24-26.
		b. Provide further relevant information on the provenance of animals, health/immune status, genetic modification status, genotype, and any previous procedures.	P. 7, lines 24-31.
**Experimental procedures**	9	For each experimental group, including controls, describe the procedures in enough detail to allow others to replicate them, including:	P. 7, lines 32-34; P. 8, lines 1-15.
		a. What was done, how it was done and what was used.	P. 8, lines 12-14; P. 23, lines 19-20.
		b. When and how often.	P. 7, lines 27-31; P. 8, lines 16-17.
		c. Where (including detail of any acclimatisation periods).	P. 4, lines 12-19; P. 7, lines 32-34.
		d. Why (provide rationale for procedures).	
**Results**	10	For each experiment conducted, including independent replications, report:	P. 12, lines 12-15; P. 13, lines 12-19; P. 23, lines 9-20.
		a. Summary/descriptive statistics for each experimental group, with a measure of variability where applicable (e.g. mean and SD, or median and range).	
		b. If applicable, the effect size with a confidence interval.	Not reported.

The Recommended Set

These items complement the Essential 10 and add important context to the study. Reporting the items in both sets represents best practice.

Item		Recommendation	Section/line number, or reason for not reporting
**Abstract**	11	Provide an accurate summary of the research objectives, animal species, strain and sex, key methods, principal findings, and study conclusions.	P. 2, lines 2-26.
**Background**	12	a. Include sufficient scientific background to understand the rationale and context for the study, and explain the experimental approach.	P. 3, lines 2-34; P. 4, lines 1-19.
		b. Explain how the animal species and model used address the scientific objectives and, where appropriate, the relevance to human biology.	Not reported.
**Objectives**	13	Clearly describe the research question, research objectives and, where appropriate, specific hypotheses being tested.	P. 2, lines 2-5; P. 4, lines 12-19.
**Ethical statement**	14	Provide the name of the ethical review committee or equivalent that has approved the use of animals in this study, and any relevant licence or protocol numbers (if applicable). If ethical approval was not sought or granted, provide a justification.	P. 8, lines 16-22; P. 19, lines 6-11.
**Housing and husbandry**	15	Provide details of housing and husbandry conditions, including any environmental enrichment.	P. 7, lines 27-31.
**Animal care and monitoring**	16	a. Describe any interventions or steps taken in the experimental protocols to reduce pain, suffering and distress.	P. 8, lines 13-22.
		b. Report any expected or unexpected adverse events.	Not reported.
		c. Describe the humane endpoints established for the study, the signs that were monitored and the frequency of monitoring. If the study did not have humane endpoints, state this.	Not reported.
**Interpretation/ scientific implications**	17	a. Interpret the results, taking into account the study objectives and hypotheses, current theory and other relevant studies in the literature.	P. 15, lines 22-34; P. 16, lines 1-34; P. 17, lines 1-34; P. 18, lines 1-7.
		b. Comment on the study limitations including potential sources of bias, limitations of the animal model, and imprecision associated with the results.	P. 18, lines 8-17.
**Generalisability/ translation**	18	Comment on whether, and how, the findings of this study are likely to generalise to other species or experimental conditions, including any relevance to human biology (where appropriate).	P. 18, lines 20-24.
**Protocol registration**	19	Provide a statement indicating whether a protocol (including the research question, key design features, and analysis plan) was prepared before the study, and if and where this protocol was registered.	Not reported.
**Data access**	20	Provide a statement describing if and where study data are available.	P. 19, lines 16-18.
**Declaration of interests**	21	a. Declare any potential conflicts of interest, including financial and non-financial. If none exist, this should be stated.	P. 19, lines 14-15.
		b. List all funding sources (including grant identifier) and the role of the funder(s) in the design, analysis and reporting of the study.	P. 19, lines 12-13.
