# Supplementary material for: ARHGAP40 suppresses clear cell renal cell carcinoma progression and is associated with altered glutamine-related metabolism and redox balance
Source: Cancer Biol Ther. 2026 Oct 8;27(1):2746137. doi: 10.1080/15384047.2026.2746137 (PMC13652080; doi:10.1080/15384047.2026.2746137)
Supplement: Supplementary file 2 [file KCBT_A_2746137_SM8485.docx]

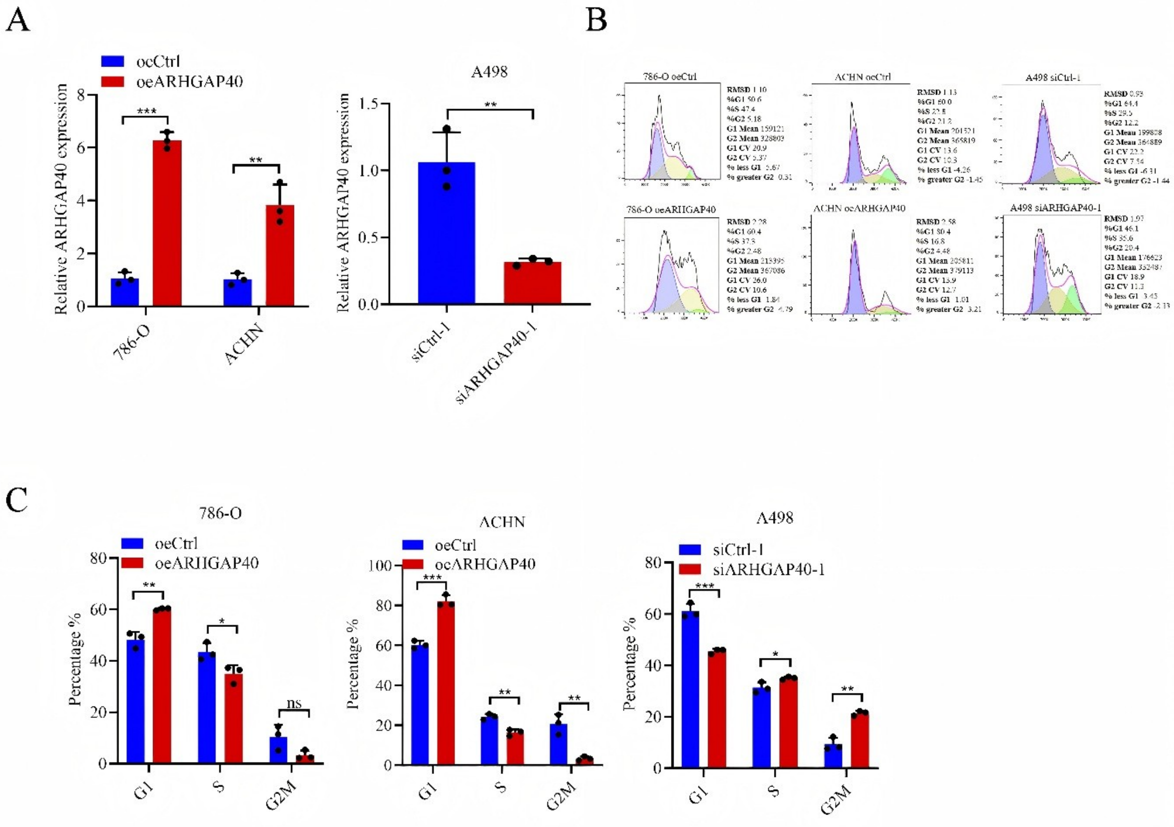

A
oeCtrl
oeARHGAP40
Relative ARHGAP40 expression
***
**
786-O
ACHN
A498
siCtrl-1
siARHGAP40-1
B
786-O oeCtrl
ACHN oeCtrl
A498 siCtrl-1
786-O oeARHGAP40
ACHN oeARHGAP40
A498 siARHGAP40-1
C
786-O
ACHN
A498
Percentage %
G1
S
G2M
ns
