# Supplementary material for: ARHGAP40 suppresses clear cell renal cell carcinoma progression and is associated with altered glutamine-related metabolism and redox balance
Source: Cancer Biol Ther. 2026 Oct 8;27(1):2746137. doi: 10.1080/15384047.2026.2746137 (PMC13652080; doi:10.1080/15384047.2026.2746137)
Supplement: Supplementary file 3 [file KCBT_A_2746137_SM8486.docx]

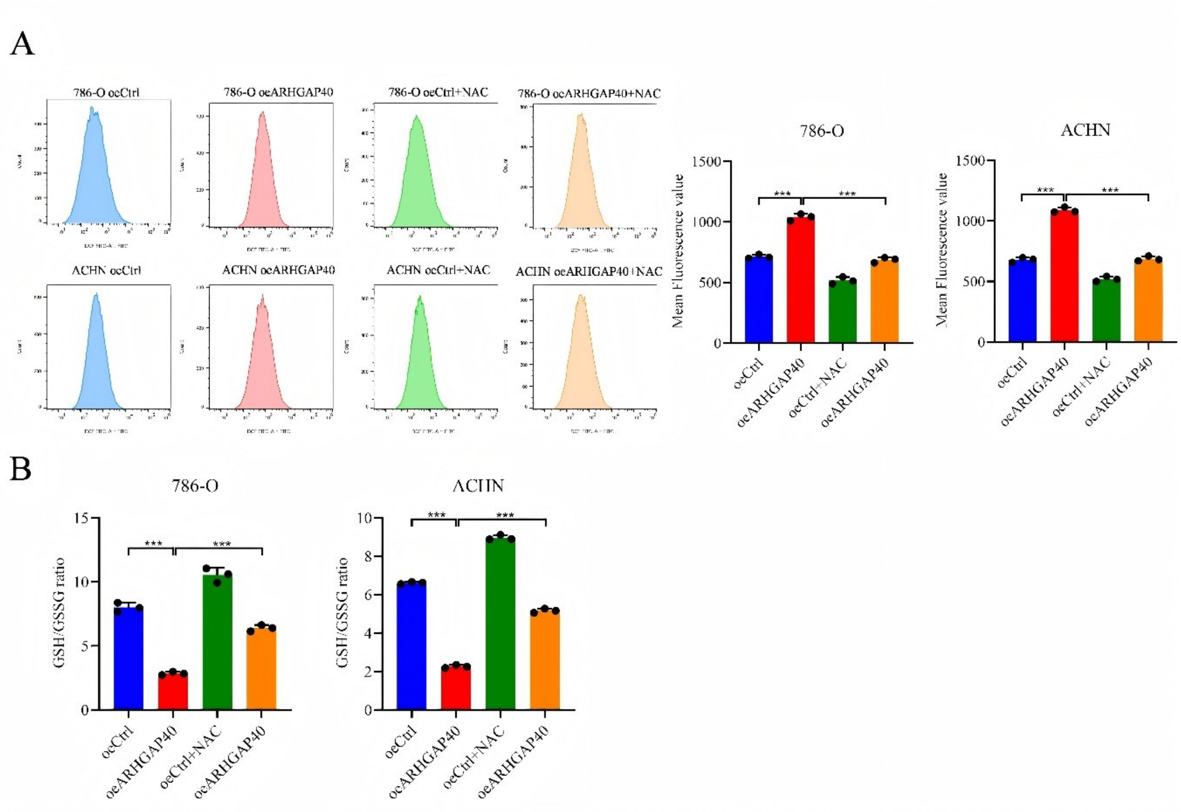
A
786-O oeCtrl
786-O oeARHGAP40
786-O oeCtrl+NAC
786-O oeARHGAP40+NAC
ACHN oeCtrl
ACHN oeARHGAP40
ACHN oeCtrl+NAC
ACHN oeARHGAP40+NAC
786-O
ACHN
Mean Fluorescence value
0
500
1000
1500
***
oeCtrl
oeARHGAP40
oeCtrl+NAC
oeARHGAP40
B
786-O
ACHN
GSH/GSSG ratio
0
5
10
15
0
2
4
6
8
10
***
oeCtrl
oeARHGAP40
oeCtrl+NAC
oeARHGAP40
