# Supplementary material for: ARHGAP40 suppresses clear cell renal cell carcinoma progression and is associated with altered glutamine-related metabolism and redox balance
Source: Cancer Biol Ther. 2026 Oct 8;27(1):2746137. doi: 10.1080/15384047.2026.2746137 (PMC13652080; doi:10.1080/15384047.2026.2746137)
Supplement: Supplementary file 4 [file KCBT_A_2746137_SM8487.docx]

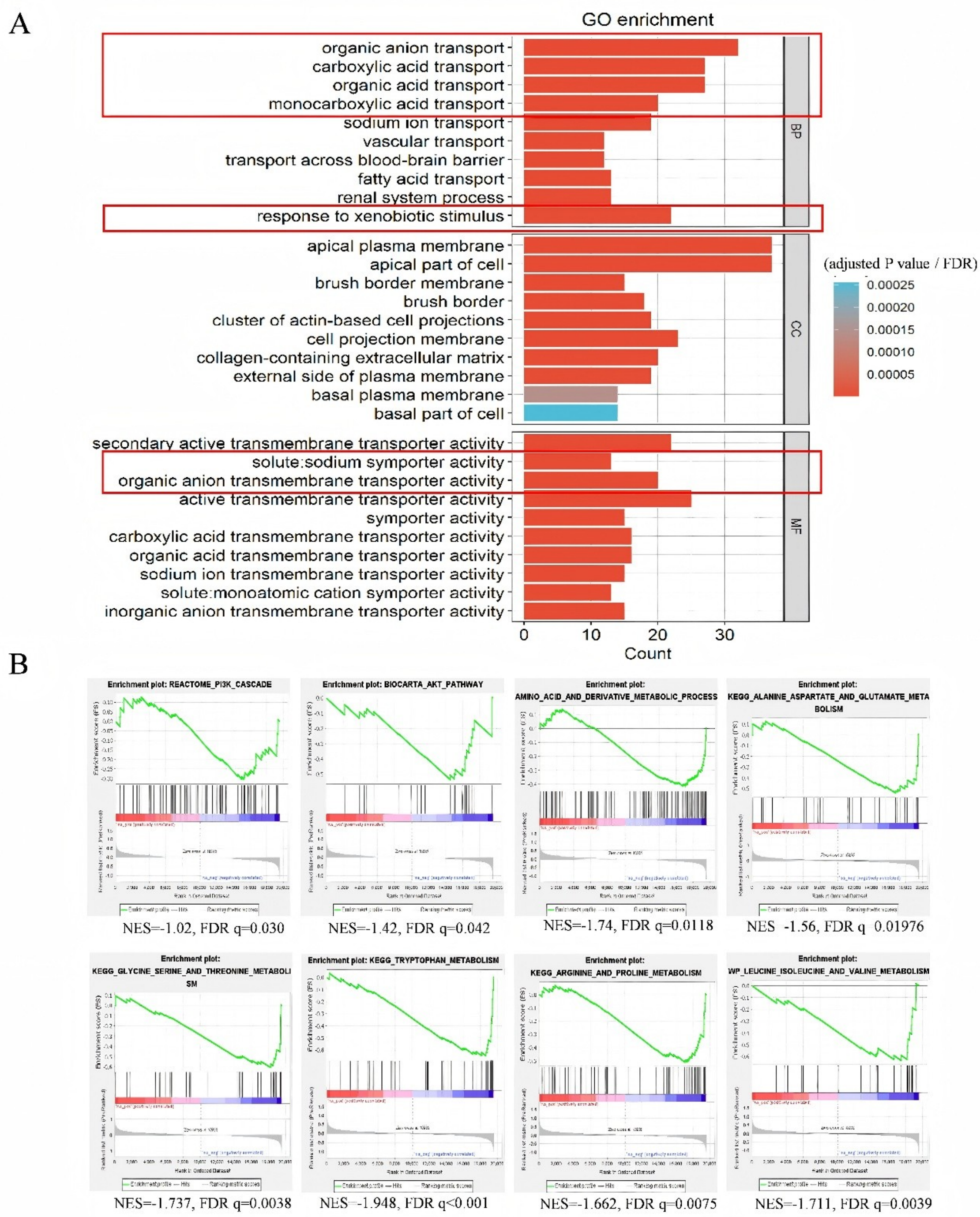
A
GO enrichment
organic anion transport
carboxylic acid transport
organic acid transport
monocarboxylic acid transport
sodium ion transport
vascular transport
transport across blood-brain barrier
fatty acid transport
renal system process
response to xenobiotic stimulus
apical plasma membrane
apical part of cell
brush border membrane
brush border
cluster of actin-based cell projections
cell projection membrane
collagen-containing extracellular matrix
external side of plasma membrane
basal plasma membrane
basal part of cell
secondary active transmembrane transporter activity
solute:sodium symporter activity
organic anion transmembrane transporter activity
active transmembrane transporter activity
symporter activity
carboxylic acid transmembrane transporter activity
organic acid transmembrane transporter activity
sodium ion transmembrane transporter activity
solute:monoatomic cation symporter activity
inorganic anion transmembrane transporter activity
BP
CC
MF
0
10
20
30
Count
(adjusted P value / FDR)
0.00025
0.00020
0.00015
0.00010
0.00005
B
Enrichment plot: REACTOME_PI3K_CASCADE
NES=-1.02, FDR q=0.030
Enrichment plot: BIOCARTA_AKT_PATHWAY
NES=-1.42, FDR q=0.042
Enrichment plot: AMINO_ACID_AND_DERIVATIVE_METABOLIC_PROCESS
NES=-1.74, FDR q=0.0118
Enrichment plot: KEGG_ALANINE_ASPARTATE_AND_GLUTAMATE_METABOLISM
NES -1.56, FDR q 0.01976
Enrichment plot: KEGG_GLYCINE_SERINE_AND_THREONINE_METABOLISM
NES=-1.737, FDR q=0.0038
Enrichment plot: KEGG_TRYPTOPHAN_METABOLISM
NES=-1.948, FDR q<0.001
Enrichment plot: KEGG_ARGININE_AND_PROLINE_METABOLISM
NES=-1.662, FDR q=0.0075
Enrichment plot: WP_LEUCINE_ISOLEUCINE_AND_VALINE_METABOLISM
NES=-1.711, FDR q=0.0039
