# Supplementary material for: ARHGAP40 suppresses clear cell renal cell carcinoma progression and is associated with altered glutamine-related metabolism and redox balance
Source: Cancer Biol Ther. 2026 Oct 8;27(1):2746137. doi: 10.1080/15384047.2026.2746137 (PMC13652080; doi:10.1080/15384047.2026.2746137)
Supplement: Supplementary file 5 [file KCBT_A_2746137_SM8488.docx]

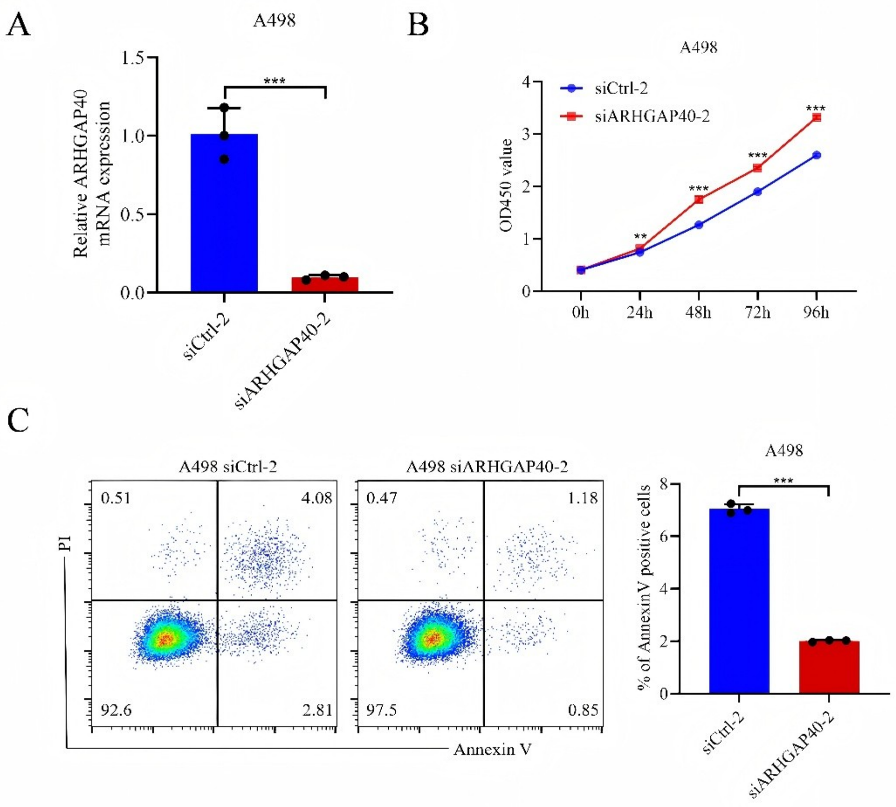
A
A498
Relative ARHGAP40 mRNA expression
1.5
1.0
0.5
0.0
***
siCtrl-2
siARHGAP40-2
B
A498
siCtrl-2
siARHGAP40-2
OD450 value
0h
24h
48h
72h
96h
C
A498 siCtrl-2
A498 siARHGAP40-2
0.51
4.08
92.6
2.81
0.47
1.18
97.5
0.85
PI
Annexin V
A498
% of Annexin V positive cells
