# Supplementary material for: ARHGAP40 suppresses clear cell renal cell carcinoma progression and is associated with altered glutamine-related metabolism and redox balance
Source: Cancer Biol Ther. 2026 Oct 8;27(1):2746137. doi: 10.1080/15384047.2026.2746137 (PMC13652080; doi:10.1080/15384047.2026.2746137)
Supplement: Supplementary file 2 [file KCBT_A_2746137_SM3143.docx]

**Supplementary Figure S1. Validation of ARHGAP40 manipulation and effects on cell-cycle distribution.**

(A) RT-qPCR validation of ARHGAP40 overexpression in 786-O and ACHN cells and siARHGAP40-1-mediated knockdown in A498 cells.
(B) Representative flow-cytometry histograms of cell-cycle distribution.
(C) Quantification of G1, S, and G2/M phase fractions.

n = 3 independent biological replicates; data are presented as mean ± SEM. Two-group comparisons were performed using two-tailed Student’s t-tests. ** *P* < 0.01, *** *P* < 0.001.

**Supplementary Figure S2. A second independent siRNA reproduces the ARHGAP40-knockdown phenotype in A498 cells.**

(A) RT-qPCR validation of ARHGAP40 knockdown by siARHGAP40-2.
(B) CCK-8 proliferation curve for siCtrl-2 and siARHGAP40-2 cells.
(C) Representative Annexin V/PI flow-cytometry plots and quantification of Annexin V-positive cells.

n = 3 independent biological replicates; data are presented as mean ± SEM. Panels A and C were analyzed using two-tailed Student's t-tests. The CCK-8 time course was analyzed by two-way repeated-measures ANOVA with Geisser-Greenhouse correction, followed by Šídák-adjusted multiple comparisons between groups at each time point. Significant effects were observed for ARHGAP40 silencing (F(1,4) = 385.89, P < 0.0001), time (F(1.64,6.57) = 14725.91, *P* < 0.0001), and the ARHGAP40 silencing × time interaction (F(1.64,6.57) = 318.31, *P* < 0.0001). ** *P* < 0.01, *** *P* < 0.001.

**Supplementary Figure S3. GO and GSEA analyses of ARHGAP40-associated transcriptional differences in TCGA-KIRC.**

(A) Gene Ontology enrichment analysis of differentially expressed genes between ARHGAP40-high and ARHGAP40-low tumors; color denotes Benjamini-Hochberg-adjusted P value/FDR.
(B) Representative GSEA plots of significantly negatively enriched gene sets, with normalized enrichment score (NES) and FDR q values shown beneath each plot.

The TCGA-KIRC tumor cohort contained n = 532 samples and was divided at the median ARHGAP40 expression level. GO terms with adjusted *P* < 0.05 and GSEA results with FDR q < 0.05 were considered significant; GSEA used 1,000 permutations.

**Supplementary Figure S4. ARHGAP40 alters extracellular glutamine consumption in ccRCC cells.**

(A) Glutamine consumption over 24 h in 786-O and ACHN cells with ARHGAP40 overexpression.
(B) Glutamine consumption over 24 h in A498 cells after ARHGAP40 knockdown. Values were normalized to cellular protein.

n = 3 independent biological replicates; data are mean ± SEM. Two-group comparisons were performed using two-tailed Student's t-test. ** *P* < 0.01, *** *P* < 0.001.

**Supplementary Figure S5. N-acetylcysteine reverses ARHGAP40-associated redox changes.**

(A) Representative ROS flow-cytometry histograms and quantification of mean fluorescence intensity.
(B) GSH/GSSG ratios. n = 3 independent biological replicates; data are mean ± SEM.

Two-way ANOVA with ARHGAP40 status and NAC treatment as factors was followed by Tukey-adjusted multiple comparisons. *** *P* < 0.001.
